# MiR-424/TGIF2-Mediated Pro-Fibrogenic Responses in Oral Submucous Fibrosis

**DOI:** 10.3390/ijms24065811

**Published:** 2023-03-18

**Authors:** Ming-Yung Chou, Pei-Ling Hsieh, Shih-Chi Chao, Yi-Wen Liao, Cheng-Chia Yu, Chang-Yi Tsai

**Affiliations:** 1 School of Dentistry, Chung Shan Medical University, Taichung 40201, Taiwan; 2 Department of Dentistry, Chung Shan Medical University Hospital, Taichung 40201, Taiwan; 3 Department of Anatomy, School of Medicine, China Medical University, Taichung 404333, Taiwan; 4 Institute of Oral Sciences, Chung Shan Medical University, Taichung 40201, Taiwan; 5 Department of Medical Research and Education, Lo-Hsu Medical Foundation, Lotung Poh-Ai Hospital, Yilan 265, Taiwan; 6 Department of Medical Research, Chung Shan Medical University Hospital, Taichung 40201, Taiwan; 7 Department of Otorhinolaryngology-Head and Neck Surgery, Changhua Christian Hospital, Changhua 500, Taiwan

**Keywords:** oral submucous fibrosis, miR-424, TGIF2

## Abstract

Oral submucous fibrosis (OSF) has been recognized as a potentially malignant disorder and is characterized by inflammation and the deposition of collagen. Among various regulators of fibrogenesis, microRNAs (miR) have received great attention but the detailed mechanisms underlying the miR-mediated modulations remain largely unknown. Here, we showed that miR-424 was aberrantly overexpressed in OSF tissues, and then we assessed its functional role in the maintenance of myofibroblast characteristics. Our results demonstrated that the suppression of miR-424 markedly reduced various myofibroblast activities (such as collagen contractility and migration ability) and downregulated the expression of fibrosis markers. Moreover, we showed that miR-424 exerted this pro-fibrosis property via direct binding to TGIF2, an endogenous repressor of the TGF-β signaling. In addition, our findings indicated that overexpression of miR-424 activated the TGF-β/Smad pathway, leading to enhanced myofibroblast activities. Altogether, our data revealed how miR-424 contributed to myofibroblast transdifferentiation, and targeting the miR-424/TGIF2 axis may be a viable direction for achieving satisfactory results from OSF treatment.

## 1. Introduction

Oral cancer is one of the most common types of cancer worldwide, and it has a poor prognosis [1]. It is estimated that there were approximately 100,000 new cases in 2020 [2]. Among various precancerous conditions, the malignant potential of oral submucous fibrosis (OSF) has been revealed to be around 7–13% [3]. Juxta-epithelial inflammation and progressive collagen deposition, along with epithelial atrophy, are common histopathologic features of OSF, which may lead to difficulty in mouth opening. Therefore, the development of effective treatments for OSF is desirable so that patients may achieve a better quality of life and so that progression toward malignancy can be managed.

The pathogenesis of OSF is multifactorial, and variations in exposure to behavioral and environmental risk factors results in variation in the incidence. The habit of chewing areca nut has long been recognized as an etiological factor [4], and the constituent of areca nuts was found to induce myofibroblast transdifferentiation from human buccal mucosal fibroblasts (BMFs) [5]. Various types of pathological fibrosis are related to the activation of resident fibroblasts and epithelial–mesenchymal transition (EMT) [6,7], and areca nut components have been shown to elicit myofibroblasts’ differentiation through activation of the TGF-β pathway [8,9] and modulation of EMT-associated factors [5]. For instance, it has been demonstrated that arecoline (an areca nut alkaloid) induces Slug expression in BMFs, which contributes to myofibroblast transdifferentiation and the upregulation of type I collagen, as Slug was able to bind to the E-box of type I collagen [10]. Constituents of areca nut not only elicited the TGF-β pathway, but they also increased oxidative stress in oral cells [11]. In fact, TGF-β and oxidative stress have been shown to promote each other and contribute to fibrogenesis [12]. Accumulation of oxidative stress further exacerbates fibrosis by inducing myofibroblast activation [13,14]. Therefore, the exploration of key regulators of myofibroblast activation is crucial in terms of the amelioration of OSF.

MicroRNAs (miRs) are small (~19–24 nucleotide) and single-stranded non-coding RNAs that function as posttranscriptional regulators by binding to complementary target sites in the three prime untranslated region (3′UTR) of target mRNAs [15]. Previously, miR-424 has been shown to be increased by TGF-β and induce the expression of myofibroblast marker, α-smooth muscle actin (α-SMA), during the EMT of lung epithelial cells [16] or myofibroblast differentiation of lung fibroblasts [17]. On the other hand, miR-424 has been shown to modulate anti-oxidant enzyme and oxidative stress markers in the peri-infarct cortex in a murine model of transient cerebral ischemia [18]. Additionally, miR-424 was found to be upregulated in oral cancer tissues [19]. Nevertheless, the significance of miR-424 in the development of precancerous OSF remains unknown, and whether miR-424 regulates myofibroblasts activities in OSF is worthy of investigation.

As such, we examined the expression of miR-424 in OSF tissues and the functional role of miR-424 in myofibroblast activities and the production of reactive oxygen species (ROS). Moreover, we identified a putative target of miR-424 and verified that miR-424 exerted its regulatory effect on myofibroblast activities by inhibiting it.

## 2. Results

### 2.1. MiR-424 Is Aberrantly Overexpressed in OSF Tissues and Positively Associated with Several Fibrosis-Associated Molecules

**Figure 1:**
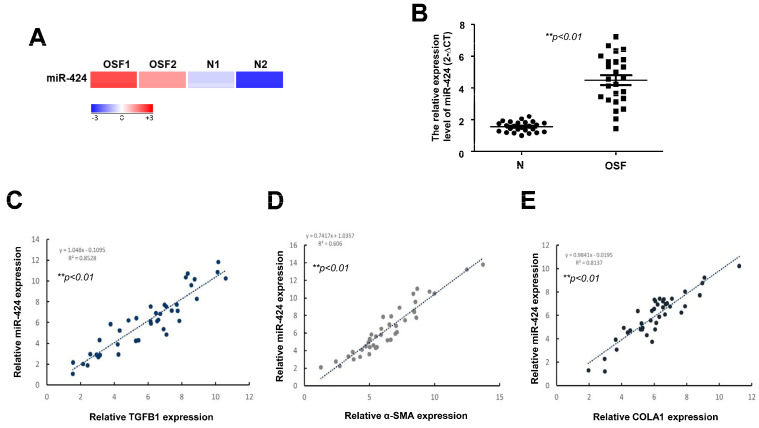
MiR-424 is upregulated in OSF tissues and positively correlated with various fibrosis-associated markers. (**A**) Heatmap showing differentially expressed miR-424 between normal control (N) and OSF samples. (**B**) qRT-PCR quantification of expression level of miR-424 in normal control and OSF tissues (n = 25). A positive correlation was observed between miR-424 and numerous fibrosis markers, including transforming growth factor beta 1(TGFB1) (**C**), alpha smooth muscle actin (ACTA2) (**D**), and alpha-1 type I collagen (COL1A1) (**E**).

First of all, RNA high-throughput sequencing analysis of OSF and normal mucosal samples indicated that miR-424 may be differentially expressed under this precancerous condition (Figure 1A). The upregulation of miR-424 in OSF specimens was validated using qRT-PCR compared to normal mucosal tissues (Figure 1B). In addition, the results of Pearson correlation measurements showed that there was a positive correlation between miR-424 and several fibrosis-related markers, including TGF-β1 (TGFB1) (C), α-SMA (ACTA2), and alpha-1 type I collagen (COL1A1) (Figure 1C–E) using qRT-PCR analysis.

### 2.2. Inhibition of MiR-424 in Fibrotic Buccal Mucosal Fibroblasts (fBMFs) Reduces Myofibroblast Activities

**Figure 2:**
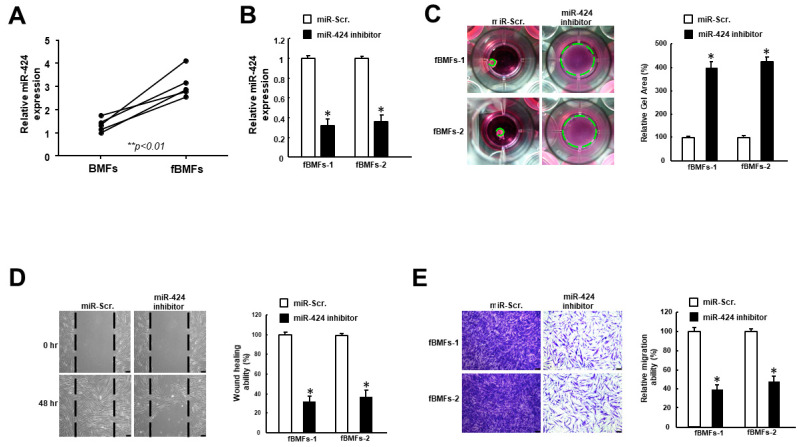
The downregulation of miR-424 in fibrotic buccal mucosal fibroblasts (fBMFs) diminishes several myofibroblast phenotypes. (**A**) The expression level of miR-424 was elevated in fBMFs compared to BMFs. (**B**) The expression of miR-424 was markedly reduced using miR-424 inhibitor. A number of myofibroblast characteristics, including (**C**) collagen gel contraction, (**D**) wound healing, and (**E**) transwell migration capacities, were all suppressed in fBMFs receiving miR-424 inhibitor. * *p* < 0.05 compared to miR-Scr. group.

Fibrotic buccal mucosal fibroblasts (fBMFs) derived from OSF tissues are commonly used to study myofibroblast features [20]. As shown in Figure 2A, the expression of miR-424 was increased in fBMFs compared to normal BMFs. In order to examine the role of miR-424 in myofibroblast activities, an inhibitor was applied to downregulate the expression of miR-424 (Figure 2B). Upon tissue injury, myofibroblasts are known to migrate to the wound area and exert contraction forces to close it, which is associated with the formation of stress fiber, such as alpha-smooth muscle actin (α-SMA) [21]. Accordingly, the subsequent experiments were conducted in order to assess myofibroblast activation. We showed that collagen gel contraction (Figure 2C), wound healing capacity (Figure 2D), and transwell migration ability (Figure 2E) were all reduced when the expression of miR-424 was inhibited.

### 2.3. MiR-424 Is Associated with ROS Accumulation and Expression of Myofibroblast Marker

**Figure 3:**
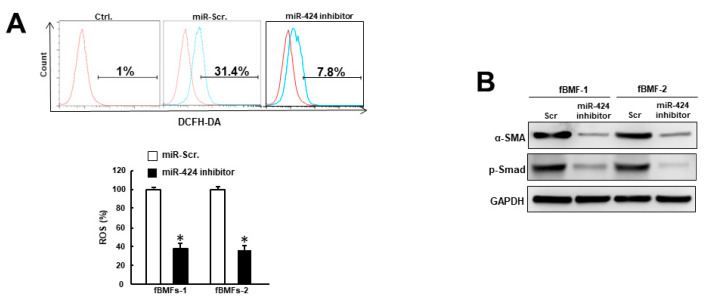
Repression of miR-424 in fBMFs decreases oxidative stress and the expression of fibrosis markers. (**A**) Intracellular reactive oxygen species (ROS) generation was decreased in fBMFs with miR-424 inhibitor using the 2′,7′-dichlorodihydrofluorescein diacetate (DCFH-DA) method. (**B**) The expression levels of α-SMA and phosphorylated Smad in fBMFs were inhibited when miR-424 was suppressed. * p < 0.05 compared to miR-Scr. group.

Given that oxidative stress confers to myofibroblast transdifferentiation [13,14] and is under the regulation of miR-424 [18], we assessed the production of reactive oxygen species (ROS) in fBMFs with miR-424 inhibitor. As expected, ROS generation was decreased when miR-424 was downregulated (Figure 3A). Additionally, we showed that the expression levels of the myofibroblast marker, α-SMA, and the phosphorylation of Smad were both inhibited after the suppression of miR-424 (Figure 3B). Collectively, these findings demonstrated that miR-424 can modulate oxidative stress and mediate myofibroblast activation.

### 2.4. MiR-424 Modulates Myofibroblast Activities through Directly Binding to TGIF2

**Figure 4:**
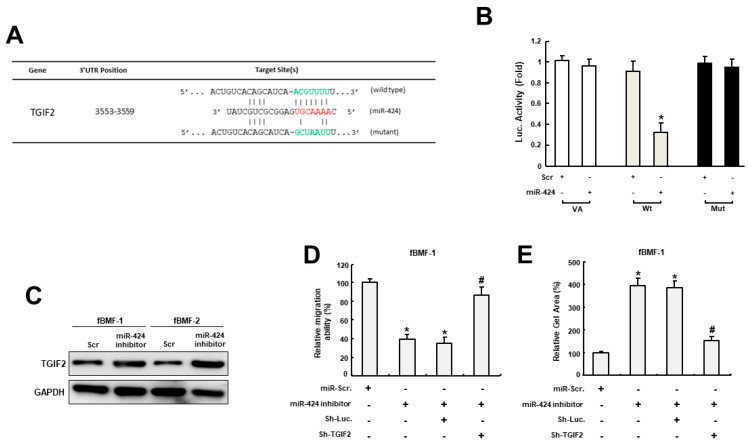
MiR-424 mediates myofibroblast activities via direct binding to TGIF2. (**A**) An illustration of the three prime untranslated regions (3′-UTR) of full-length (wild type) and mutated TGIF2 complementarity between the seed sequence of miR-424 predicted by the TargetScan in silico browser. (**B**) The luciferase activity of the reporter construct containing the wild-type TGIF2 3′-UTR was repressed by miR-424, whereas there was no effect on the activity of reporter constructs containing mutant TGIF2 3′-UTR in BMFs. (**C**) The expression of TGIF2 was upregulated in fBMFs with miR-424 inhibitors. The effects of the miR-424 inhibitor on migration capacity (**D**) and collagen gel contractility (**E**) were reversed in fBMFs when the expression of TGIF2 was reduced. * *p* < 0.05 compared to miR-Scr. group; # *p* < 0.05 compared to miR-424 inhibitor+Sh-Luc. group.

In order to unveil the target gene of miR-424, we used bioinformatics software as a means to predict the putative targets of miR-424 and found that TGFB-induced factor 2 protein (TGIF2) contained a potential binding site for miR-424 (Figure 4A). The following luciferase reporter assay further demonstrated that luciferase activity was markedly reduced in cells co-transfected with miR-424 and wild-type TGIF2, while there was no change in the mutant group (Figure 4B). Additionally, the expression of TGIF2 was increased in fBMFs treated with miR-424 inhibitor (Figure 4C). Furthermore, the suppression of migration ability by miR-424 inhibitor was reversed in fBMFs transfected with sh-TGIF2 (Figure 4D). Similarly, the effect of miR-424 on the suppression of collagen gel contraction in fBMFs was counterbalanced after the silencing of TGIF2. These results suggested that miR-424 mediates various myofibroblast activities by directly binding to TGIF2 and reducing its expression. 

### 2.5. MiR-424 Induces Myofibroblast Transdifferentiation via Activation of TGF-β Signaling

**Figure 5:**
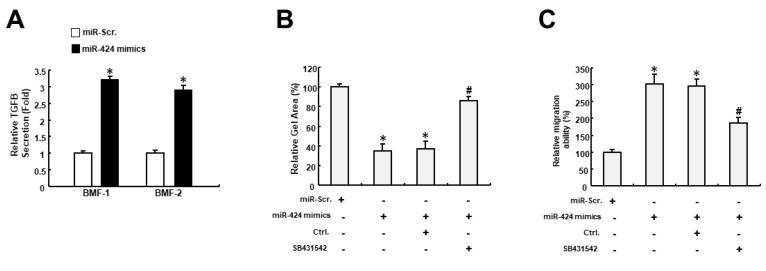
The miR-424-induced myofibroblast features may result from the activation of the TGF-β pathway. (**A**) The secretion of TGF-β was increased in BMFs treated with miR-424 mimics. (**B**) Collagen gel contractility was increased in miR-424 mimics-receiving cells, while inhibition of the TGF-β pathway using SB431542 prevented it. (**C**) The increased migration ability elicited by miR-424 mimics was reduced by SB431542. * *p* < 0.05 compared to miR-Scr. group; # *p* < 0.05 compared to miR-424 mimics+ Ctrl. group.

As TGFIF2 is a transcriptional repressor that can interact with TGF-β-activated SMAD proteins, we examined whether miR-424 affected TGF-β activation. As shown in Figure 5A, the expression of TGF-β secretion was upregulated in BMFs receiving miR-424 mimics. Further, we demonstrated that the miR-424-induced collagen gel contractility of BMFs was abrogated by inhibition of the TGF-β pathway using SB431542 (Figure 5B). Likewise, the increased migration capacity of miR-424 mimics-treated BMFs was downregulated when SB431542 was applied (Figure 5C). Taken together, these results showed that miR-424 possesses the ability to induce myofibroblast transdifferentiation through the activation of TGF-β as a result of the direct suppression of TGFIF2.

## 3. Discussion

In this study, we demonstrated that miR-424 was aberrantly upregulated in OSF tissues and fBMFs. Our results showed that suppression of miR-424 markedly inhibited myofibroblast activation, including myofibroblast activities and the expression of myofibroblast marker α-SMA. MiR-424 has been found to be overexpressed in idiopathic pulmonary fibrosis (IPF) tissues [16,17] and TGF-β1-treated human lung fibroblasts [17]. It has been shown that the TGF-β1-induced upregulation of miR-424 requires the canonical TGF-β1 signaling pathway and that increased miR-424 further enhances the expression of α-SMA in human alveolar basal epithelial cells [16] and the TGF-β1-induced myofibroblast differentiation of lung fibroblasts [17]. These studies revealed that miR-424 not only responded to TGF-β1 stimulation, but that it also potentiated TGF-β signaling through directly binding to various negative regulators of the TGF-β pathway, such as Smad ubiquitin regulatory factor 2 (Smurf2) [16] and slit guidance ligand 2 (Slit2) [16]. Smurf2 has been found to ubiquitinate TGF-β receptors and Smad proteins, leading to proteasomal degradation [22]. Slit2 has been proven to reduce TGF-β-induced collagen synthesis and Smad2/3 transcriptional activity in renal fibroblasts [23]. In line with these findings, we showed that miR-424 promoted myofibroblast activities by inhibiting TGIF2.

TGIF2 belongs to the three-amino-acid-loop extension (TALE) superfamily of homeodomain proteins and acts as a transcriptional repressor by recruiting histone deacetylases to TGF-β-responsive genes and interacting with TGF-β-activated Smad3 [24]. It has been demonstrated that the repression of histone deacetylase simulates the expression of TGIF2, which is associated with reduced TGF-β-induced α-SMA expression in skin fibroblasts [25] and human corneal fibroblasts [26]. TGIF2 also participates in the TGF-β-evoked transdifferentiation of fibroblasts under the modulation of miRs. It has been shown that overexpressed miR-34a increases the expression of α-SMA, collagen I, TGF-β1, and p-Smad2/3 in intrahepatic biliary epithelial cells by targeting TGIF2 [27]. Here, we demonstrated that TGIF2 was regulated by miR-424, and it was involved in the miR-424-mediated myofibroblast activation of fBMFs. We also showed that the secretion of TGF-β was upregulated in BMFs with forced expression of miR-424. Further, miR-424-induced myofibroblast activation required the activation of the TGF-β pathway, as the effects of miR-424 mimics were abrogated by the inhibitor of TGF-β signaling. Our results showed that overexpressed miR-424 promoted myofibroblast transdifferentiation by enhancing the production of TGF-β and inhibiting the TGF-β pathway via direct binding to TGIF2.

It is well known that TGF-β stimulates ROS production, which contributes to pathological fibrosis [12]. Therefore, it comes as no surprise that various oxidative stress biomarkers, such as malondialdehyde or 8-hydroxy-2-deoxyguanosine, were upregulated in OSF [28]. In an effort to evaluate the role of miR-424 in oxidative stress accumulation, an miR-424 inhibitor was applied, and we showed that ROS generation was suppressed. Our findings were consistent with another study showing that miR-424 conferred to the LPS-induced ROS production in lipopolysaccharide (LPS)-treated pulmonary alveolar epithelial cells [29] or LPS-stimulated proximal tubule epithelial cells [30]. However, miR-424 was revealed to abrogate oxidative stress-associated injury by increasing antioxidant enzymes and reducing oxidative stress indicators in C57/BL6 mice with transient cerebral ischemia [18]. These results indicated that miR-424 may exhibit pro- or anti-fibrosis properties under various disease conditions.

**Figure 6:**
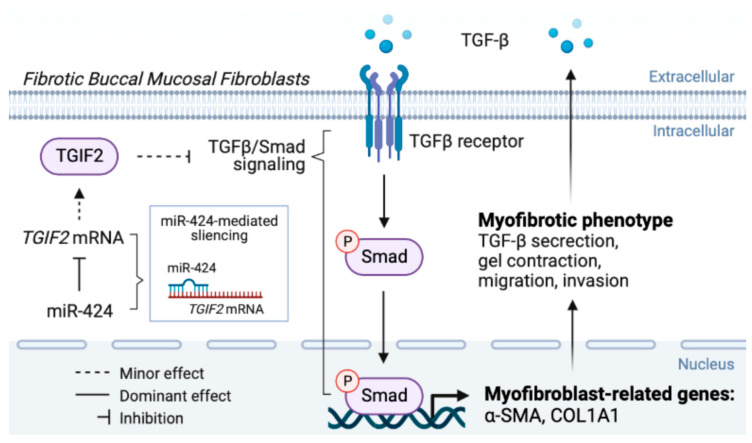
The graphic abstract of the present study illustrates the mechanisms of the miR-424/TGIF2 axis in the regulation of myofibroblast transdifferentiation in oral submucous fibrosis.

Altogether, current evidence and our findings suggested that there is a feed-forward loop between TGF-β signaling and miR-424 which promotes and maintains myofibroblast activation and collagen deposition. Our data demonstrated that miR-424 can induce another contributor of myofibroblast transdifferentiation, ROS accumulation, which amplifies pathological fibrosis in the oral cavity. Accordingly, targeting the miR-424/TGIF2 axis may be an effective approach to ameliorate fibrosis progression in OSF patients (Figure 6).

## 4. Materials and Methods

### 4.1. Tissue Collection, Cell Culture, and Reagents

Specimens of normal and fibrotic buccal mucosa were respectively collected from healthy and OSF individuals undergoing resection at the Department of Dentistry, Chung Shan Medical University Hospital according to a procedure approved by the Institutional Review Board in Chung Shan Medical University Hospital, Taichung, Taiwan, and informed written consent was obtained from each individual.

Normal buccal mucosal fibroblasts (BMFs) and fibrotic buccal mucosal fibroblasts (fBMFs) were respectively obtained from the normal and fibrotic buccal mucosa specimens of the same patients. After being washed with phosphate-buffered saline (PBS), tissues were minced into 1 mm^2^-sized pieces and incubated with Trypsin-EDTA (0.05%) for 30 to 60 min. The supernatants were removed after centrifugation at 1200 rpm for 5 min; the tissue pellets were suspended in cultured medium (Dulbecco’s Modified Eagle’s Medium containing 10% [*v/v*] fetal bovine serum and 1% [*v/v*] penicillin–streptomycin). Cells with a spindle-shaped morphology that migrated from the tissue were identified as fibroblasts. All cells were routinely maintained in culture medium at 5% CO_2_ at 37 °C, and cells in the third to eighth passages were used in subsequent experiments [31]. The TGF-β-Smad inhibitor (SB-431542) and all other reagents used in this study were purchased from Sigma-Aldrich (St. Louis, MO, USA) unless otherwise noted.

### 4.2. Quantitative Real-Time PCR (qRT-PCR)

For the preparation of clinical specimens, tissues excised immediately from the surgery were placed in liquid nitrogen and stored, frozen, at −80 °C. Total RNA was prepared from tissues and cells using Trizol reagent according to the manufacturer’s protocol (Invitrogen Life Technologies, Carlsbad, CA, USA). QRT–PCRs of mRNAs were reverse-transcribed using the Superscript III first-strand synthesis system for RT–PCR (Invitrogen Life Technologies, Carlsbad, CA, USA). QRT-PCR reactions with the resulting cDNAs were performed on an ABI StepOne™ Real-Time PCR System (Applied Biosystems). The primer sequences are listed below: α-SMA, 5′-AGCACATGGAAAAGATCTGGCACC-3′ (forward), and 5′-TTTTCTCCCGGTTGGCCTTG-3′ (reverse); COL1A1, 5′-GGGTGACCGTGGTGAGA-3′ (forward) and 5′-CCAGGAGAGCCAGAGGTCC-3′ (reverse); GAPDH, 5′-CTCATGACCACAGTCCATGC-3′ (forward), and 5′-TTCAGCTCTGGGATGACCTT-3’ (reverse). Pearson’s correlation analysis between miR-424 expression and fibrotic genes’ levels were determined by qRT-PCR analysis [32].

### 4.3. MiRNAs Mimic and Inhibition

Synthesized oligonucleotides of miR-424 mimic, miR-424 inhibitor, and miR-scramble were purchased from ThermoFisher Scientific (Carlsbad, CA, USA). In order to overexpress and inhibit the endogenous miR-424, miR-424 mimic and miR-424 inhibitor were transfected into cells, respectively, using Lipofectamine 2000 in accordance to the manufacturer’s instructions (LF2000, Invitrogen, Carlsbad, CA, USA), while the miR-scramble was used as a negative control. For the detection of miR-424 levels, qRT–PCR was performed using TaqMan miRNA assays with specific primer sets (Applied Biosystems, Carlsbad, CA, USA) [33].

### 4.4. Collagen Contraction Assay

Cells were suspended in 0.5 mL of 2 mg/mL collagen solution and added to one well of a 24-well plate. The plate was incubated at 37 °C for 2 h, which caused the polymerization of collagen cell gels. After detaching gels from wells, the gels were further incubated in 0.5 mL medium for 48 h. Contraction of the gels was photographed and measured using ImageJ software (NIH, Bethesda, MD, USA) to calculate their areas [34].

### 4.5. Wound Healing Assay

Once cell confluence reached 80% in a 12-well culture plate, a denuded area was created by scratching the cell monolayer with a sterile 200 µL pipette tip across the center of the well. After another 48 h of incubation, cells were stained with crystal violet, allowing for visualization under a microscope. Cell movement towards the wound area was photographed at 0 and 48 h [35].

### 4.6. Transwell Migration Assays

Cells were placed in the upper chamber of a transwell (Corning, Acton, MA, USA) with serum-free medium. Medium containing 10% fetal bovine serum was added to the lower chamber as a chemo-attractant. After another incubation for 24 h, cells attached to the other side of the membrane were stained with crystal violet and were counted in five randomly selected fields under microscopy.

### 4.7. Reactive Oxygen Species Production Analysis

ROS production was assessed using flow cytometry as the fluorescence of 2′,7′-dichlorofluorescein (DCF) which are the oxidation products of 2′,7′-dichlorodihydrofluorescein diacetate (DCFH-DA) with a sensitivity for H_2_O_2_/NO-based radicals. Cells were incubated with 10 μM DCFH-DA for 60 min at 37 °C and were then washed twice with PBS. The DCF fluorescence of 10,000 cells was analyzed by flow cytometry (Becton Dickinson, Mountain View, CA, USA) at excitation and emission wavelengths of 488 and 525 nm, respectively [36].

### 4.8. Western Blotting

Whole-cell lysates were obtained by using NP-40 lysis buffer containing 50 mM Tris-HCl (pH 7.4), 150mM NaCl, 1%NP-40, and 5 mM EDTA (ThermoFisher Scientific, Carlsbad, CA, USA). Twenty-five μg of total protein of whole-cell lysates were separated by 10% SDS-PAGE electrophoresis and were then transferred onto a polyvinylidene difluoride (PVDF) membrane (Millipore, Billerca, MA, USA) The membranes sequentially underwent blocking with 5% bovine serum albumin (BSA) in TBST (Tris-buffered saline with 0.1% Tween-20) and incubation with TGIF2 antibody (Abcam, Cambridge, UK) and the corresponding secondary antibody. The ECL-plus chemiluminescence substrate (Millipore, Billerica, MA, USA) was used for the development of the signals of immunoreactive bands, which were captured using a LAS-1000plus Luminescent Image Analyzer (GE Healthcare Biosciences, Piscataway, NJ, USA) and were quantified using ImageJ software (NIH, Bethesda, MD, USA). All antibodies were purchased from ThermoFisher Scientific (Carlsbad, CA, USA).

### 4.9. MiRNA-Targeting Gene Prediction and Dual-Luciferase Reporter Assay

The wild-type TGIF2-3’UTR was cloned into the β-gal control plasmid according to the manufacturer’s protocol. The mutant reporter was generated by replacing the original sequence ACGUUUU in the wild-type reporter with GCUAAUU. The ß-galactosidase activity of vector-only plasmid, the wild-type reporter, and the mutant reporter were normalized using the luciferase activity of a co-transfected plasmid-expressing luciferase in order to represent the background reporter activity. The reporter plasmid and miR-424 mimic or miR-Scramble were co-transfected into cells using a Lipofectamine 2000 reagent (LF2000, Invitrogen, Carlsbad, CA, USA). Firefly luciferase activity, after normalizing to transfection efficiency, represented reporter activity [36].

### 4.10. Lentiviral-Mediated RNAi for Silencing TGIF2:

The pLV-RNAi vector was purchased from Biosettia Inc. (San Diego, CA, USA). The method of cloning the double-stranded shRNA sequence followed the manufacturer’s protocol. The oligonucleotide sequence of lentiviral vectors expressing shRNA that targets human *TGIF2* was synthesized and cloned into pLVRNAi in order to generate a lentiviral expression vector. The target sequences for *TGIF2* are listed as follows: Sh-TGIF2-1 5′- AAAAGCTGCCAAATTCAGTCCTATTGGATCCAATAGGACTGAATTTGGCAGC-3′. Lentivirus production was performed through the co-transfection of plasmid–DNA mixture with lentivector and helper plasmids (VSVG and Gag-Pol) into 293T cells (American Type Culture Collection, Manassas, VA, USA) using Lipofectamine 2000 (LF2000, Invitrogen, Carlsbad, CA, USA) [37].

### 4.11. ELISA Analysis

The concentration of TGF-β in culture medium was determined by ELISA kits according to the manufacturer’s protocol (R&D Systems, Minneapolis, MN). The absorbance was measured with a 450 nm filter on a microplate reader (MRX; Dynatech Laboratories, Chantilly, VA, USA) [38].

### 4.12. Statistical Analysis

Statistical Package of Social Sciences software (version 13.0) (SPSS, Inc., Chicago, IL, USA) was used for statistical analysis. Data from at least triplicate analysis were shown as mean ± SEM. Student’s *t* test was used to determine the statistical significance of the differences between experimental groups; *p* values less than 0.05 were considered statistically significant.

## Data Availability

Not applicable.

## Abbreviations

*ACTA2*	Actin alpha 2
α-SMA	Alpha-smooth muscle actin
BMFs	Buccal mucosal fibroblasts
*COL1A1*	Collagen type I alpha 1 chain
DCF	2′,7′-dichlorofluorescein
DCFH-DA	2′,7′- dichlorodihy-drofluorescein diacetate
EMT	Epithelial-mesenchymal transition
fBMF	Fibrotic buccal mucosal fibroblasts
GAPDH	Glyceraldehyde-3-phosphate dehydrogenase
MiR-424	MicroRNA-424
OSF	Oral submucous fibrosis
TGF-β1	Transforming growth factor Beta 1
TGIF2	TGFB-induced factor 2 protein
ROS	Reactive oxygen species
3′UTR	Three prime untranslated region
